# The Image of Healthcare Institutions in the Opinion of Patients—Evaluation of Factors Influencing the Assessment of Public Hospitals

**DOI:** 10.3390/healthcare14121690

**Published:** 2026-06-12

**Authors:** Janina Kulińska, Jolanta Grzebieluch

**Affiliations:** Department of Public Health, Medical University of Wroclaw, 50-367 Wroclaw, Poland; janina.kulinska@umw.edu.pl

**Keywords:** hospital image, patient satisfaction, public hospitals, health services perception, patient expectations, sociodemographic factors, patient experience, healthcare communication, Poland, health care quality

## Abstract

**Introduction**: Patients are increasingly aware of ways to manage their own health—especially regarding chronic diseases—along with the fundamental factors that should be present in well-organized and patient-oriented healthcare organizations. Due to the fact that the image of healthcare organizations depends on patients’ opinions, healthcare organizations are continuously improving and transforming their processes to increase patient satisfaction. This study aimed to analyze the relationship between patients’ opinions about the public hospitals in which they were treated and selected factors, including socio-demographic characteristics, previous hospital experiences, sources of information, and satisfaction with hospitalization in Poland. **Methods**: A cross-sectional survey was conducted among patients hospitalized in eight public hospitals in Wrocław. A self-developed questionnaire included two sections: (I) opinions about the hospital (11 items) and (II) expectations and satisfaction (12 items). Questionnaires were distributed in person. Data were analyzed using descriptive and inferential statistics, including correlation and chi-square tests. **Results**: Hospital image was shaped mainly by interpersonal factors, particularly staff kindness (82.9%), access to specialists (75.4%), and a sense of safety (54.4%). Women were more likely than men to seek information about hospitals before admission (47.6% vs. 39.3%; *p* = 0.021). A positive correlation was found between patient expectations and satisfaction with hospitalization (ρ = 0.425; *p* < 0.001). Media exposure played a minor role in shaping hospital image (22.1%), while personal recommendations and previous experience were the dominant sources of influence. **Conclusions**: Patients’ assessments of hospital image are determined primarily by relational and communication factors rather than infrastructural or technical aspects. Sociodemographic characteristics, such as gender and previous contact with the institution, may moderate these perceptions. The findings highlight the need to strengthen patient-centered care models, improve communication competencies among health professionals, and develop transparent institutional communication strategies.

## 1. Introduction

The image of healthcare institutions is becoming increasingly important in the context of the dynamic changes taking place in the modern healthcare system. These changes concern not only the organisational and management structure of healthcare facilities but also, above all, the relationships that develop between medical staff and patients. The previous paternalistic model, in which the patient remains a passive recipient of healthcare services, gradually gives way to a partnership model based on active patient participation in the treatment process, two-way communication and mutual trust [1,2]. The role of communication in improving treatment outcomes and patient satisfaction is increasingly emphasized in empirical studies, especially in the context of the elderly population [3].

In the literature on the subject, the image of a hospital is defined as a set of beliefs, feelings and impressions that a patient associates with a specific facility, including both direct experiences resulting from their own hospitalization and indirect information, such as media reports or recommendations from other people [4]. This multidimensional construct includes both rational aspects, such as the facility’s equipment or waiting times for services, and emotional aspects, such as the sense of security or empathy of the medical staff. In an era of growing social expectations and democratization of access to information, patients, who are increasingly aware of participants in the treatment process, evaluate medical facilities not only as places of therapy but also as institutions that ensure certain standards of service and social value [5,6].

International studies indicate that a positive image of a hospital can act as an intermediary between service quality and patient loyalty, strengthening patient satisfaction and willingness to use the facility’s services again [7,8]. Furthermore, it has been proven that the image of a healthcare institution plays a key role in the context of increased health awareness and the experiences of patients with chronic diseases, influencing their perception of the effectiveness and safety of care [9]. An important manifestation of the changing relationship between patients and healthcare facilities is patient empowerment, which consists of the conscious and active participation of patients in therapeutic decision-making and in managing their own health [10]. This phenomenon poses new challenges for medical facilities—the need to develop the communication skills of staff and build a positive image on the basis of open dialogue, respect and trust. Patient empowerment affects the healthcare system, in which patient involvement in decision-making processes and managing patients’ own health are important [11]. As shown by the results of studies conducted in emergency departments, the level of patient trust in nursing staff correlates with their assessment of the quality of care, which further emphasizes the importance of professional communication [12].

Previous studies indicate that the perception of the image of healthcare institutions is shaped by many factors, the key ones being the quality of interpersonal communication, the availability of healthcare services, the conditions of the premises and the overall organization of treatment [13,14]. Increasing attention has also been given to analyzing the impact of patients’ sociodemographic characteristics, such as age, gender, level of education and professional status, on the way healthcare facilities are perceived and evaluated [15]. Although several Polish studies have addressed selected aspects of patients’ opinions about healthcare, including patient satisfaction, trust in healthcare institutions, respect for patients’ rights, and perceptions of healthcare services, the patient-perceived image of public hospitals has not been comprehensively examined. In particular, limited attention has been paid to the combined role of sociodemographic characteristics, previous hospital experience, sources of information, media perception, expectations, and satisfaction in shaping the image of public hospitals in Poland. Therefore, there is still a lack of comprehensive empirical research on the specifics of these relationships in the context of the Polish healthcare system.

The aim of this study was to evaluate how selected factors, including patients’ sociodemographic characteristics, previous hospital experiences, sources of information, and satisfaction with hospitalization, are associated with the patient-perceived image of public hospitals in Poland. Although the image of a hospital is shaped by multiple stakeholders and determinants, including institutional communication, media coverage, organizational performance, staff attitudes, and the broader social context, the present study focuses specifically on the patient perspective. This approach was adopted because patients are the direct recipients of hospital services and their assessments integrate both functional aspects of care, such as accessibility and organization, and relational aspects, such as communication, empathy, trust, and perceived safety. Thus, the study focuses on the patient-perceived image of public hospitals, shaped by previous experiences, interpersonal communication, information sources, and satisfaction with hospitalization [16].

## 2. Materials and Methods

The study was conducted in eight public hospitals located in Wrocław, which allowed us to capture the local context, taking into account differences in the functioning of hospitals and patients’ expectations regarding the quality of services provided. Data were collected between October 2017 and October 2018. Inclusion criteria included admission to one of the selected public hospitals, the ability to understand the questionnaire, and consent to participate in the study. Patients were excluded if their clinical condition prevented them from participating in the study, if they were unable to understand or complete the questionnaire, or if they refused to participate. Minors did not take part in the study, nor did those who were hospitalised for less than 24 h. The self-designed questionnaire consisted of two parts: Part I—Opinion about the hospital—covering, among other things, sources of information about the healthcare system, perceived credibility of the media, factors influencing the choice of hospital, and assessment of the hospital’s characteristics and image. Part II—Expectations and satisfaction, concerning expectations of the facility, level of satisfaction with the stay, subjective well-being of patients, their relationships with staff, access to information and health promotion. Both closed-ended and open-ended questions were included in the questionnaire. Closed-ended questions were analyzed quantitatively. Responses to open-ended questions, including patients’ spontaneous associations with the hospital in which they were hospitalized, were reviewed and grouped into descriptive thematic categories, such as staff professionalism, kindness and empathy, quality of care, hospital infrastructure, cleanliness, organization, safety, atmosphere, and hospital modernity. These responses were used to support the interpretation of quantitative findings. The scale uses a five-point Likert scale (from 1—definitely not to 5—definitely yes). In addition, multiple-choice questions were included (e.g., the most important characteristics of the hospital, sources of information, and factors influencing the choice of facility).

The questionnaire was developed by the authors on the basis of a literature review and the study objective. The questionnaire included items referring both to the general perception of the healthcare system and to the patient’s direct experience with the specific hospital. The main objective of the study was to assess the patient-perceived image of public hospitals, not to evaluate the healthcare system as a whole. However, questions concerning the general healthcare system were included because the image of a specific healthcare institution may be influenced by the broader public perception of healthcare, media reports, and previous experiences with healthcare services. Questionnaire items were related to the local and institution-specific context, as patients were asked about the particular hospital in which they were currently hospitalized. These items concerned previous contact with the hospital, media information about that hospital, sources of opinions about the facility, reasons for choosing the hospital, proximity to the place of residence, waiting time for admission, and willingness to recommend the hospital.

Before the main study, a pilot survey was conducted among hospitalized patients. The pilot study indicated the need to modify some questions to improve their clarity and comprehensibility. In particular, the terminology used in selected questions was simplified to make the questionnaire more understandable for patients with different levels of education and health literacy.

The questionnaires were distributed and collected personally by the researcher in hospital wards, which increased the completeness and accuracy of the data. To reduce the risk of normative or socially desirable responses, particularly in relation to potentially sensitive topics, participation was voluntary and anonymous, and patients were informed that the researcher was not a hospital employee. This procedure was intended to limit social desirability bias, which is especially relevant in research involving sensitive issues [17]. Statistical analysis was performed via IBM SPSS Statistics 26.0. For nominal and ordinal variables, frequencies (*n*) and percentages (%) were calculated, whereas for quantitative variables, arithmetic means (*M*), standard deviations (*SD*), medians (*Me*), quartiles (*Q*1 and *Q*3), and minimum and maximum values were calculated. The normality of the distribution of variables was checked via the Shapiro–Wilk, Kolmogorov–Smirnov and Lilliefors tests. In the case of deviations from normality, nonparametric tests were used: the Mann–Whitney U test for two groups and the Kruskal–Wallis test for more than two groups. The relationships between variables were analysed via Spearman’s rank correlation coefficient (rho). Values of *p* < 0.05 were considered statistically significant.

## 3. Results

The study included patients hospitalized in eight multispecialty public hospitals with different clinical profiles. Taking into account the different profiles of the participating healthcare institutions, patients were recruited in each hospital from an internal medicine ward, a surgical ward, and a third randomly selected ward. This approach was adopted to improve comparability between hospitals. The final sample consisted of 778 hospitalized patients. The study involved 445 women (57.2%) and 333 men (42.8%) aged 15–90 years (*M* = 53.2; *SD* = 17.2). Basic statistics characterizing the surveyed patients are presented in Table 1.

Basic statistics characterizing the surveyed patients are presented in Table 1. Qualitative variables (nominal, e.g., gender, and ordinal, e.g., education level) are presented as frequencies (*n*) and proportions (%). For quantitative variables (e.g., age), the mean values (*M*), standard deviations (*SD*), medians (*Me*), lower quartiles (*Q*1), upper quartiles (*Q*3), and minimum (*Min*) and maximum (*Max*) values were calculated. An overall description of the study population is provided in Table 1.

Most respondents were women (57.2%). The average age of the patients was 53.2 years (*SD* = 17.2), and the age range was between 15 and 90 years. More than half of the participants had secondary education (37.3%) or higher education (35.3%). The most numerous group consisted of professionally active patients (46.4%) and pensioners (42.5%). Most respondents lived in Lower Silesia (91.0%), of whom 50.4% lived in Wrocław itself. Patients stayed in hospitals most often for 2–3 days (31.0%) or a week or longer (29.6%). For more than half of the respondents (57.2%), it was their first stay in a given hospital. The patients were asked where they obtained information about the healthcare system and whether they were interested in this topic. The most common sources of information about the healthcare system were the Internet (51.9%), acquaintances and friends (43.8%) and television (41.6%). A total of 32.3% of the respondents declared their interest in information about the healthcare system as ‘moderate’, and 17.6% followed it regularly. Women showed a higher level of interest than men did (*p* < 0.05). The respondents indicated that the media most often convey negative and sensational information about the healthcare system, much less often emphasizing its positive aspects (Table 2).

The respondents indicated that the media most often convey negative health-related information, such as disasters and accidents (39.5%), financial problems of the system (36.9%) and protests by medical professionals (34.6%). They were less likely to notice positive messages. Women noticed information about disasters more often than men did (*p* = 0.009), while the overall level of interest in healthcare issues was also significantly greater among women (*p* = 0.049). The credibility of the media was assessed cautiously—only 7% considered it completely credible, and 28.3% found it difficult to assess. Most patients assessed healthcare functioning negatively (33.3%), with only 15% assessing it positively. Men gave a slightly higher rating than women did (*p* = 0.045).

The study examined what most influenced their choice of hospital. The most important factor in choosing a hospital was the opinion of family and friends (34.1%), followed by the proximity of the facility (22.6%) and short waiting times for admission (20.1%). Nineteen percent of patients cited their own previous experiences. Only 5.7% of the respondents admitted that their choice was motivated by media information (Table 3).

**Figure 1:**
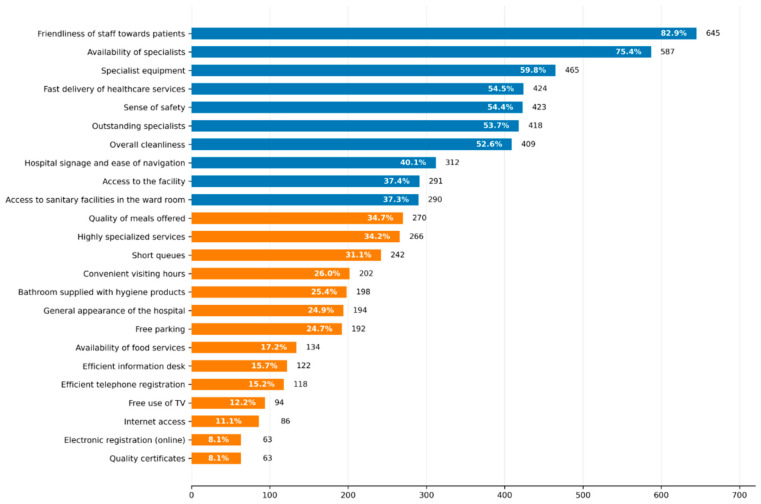
Factors influencing patient satisfaction with treatment.

Patients were asked whether they had ever been interested in their opinions about the facility before. As many as 44.1% of patients had previously searched for opinions about the hospital where they later found themselves. This difference was related to sex (*p* < 0.05). Women were more likely to seek information (47.6% vs. 39.3%; *p* = 0.021). The starting point for planning and implementing activities related to creating the image of a medical facility is to identify the areas that are most valuable to the patient. The respondents were asked to indicate up to ten of the most important elements that, regardless of the specific facility, influence their perception of the quality of treatment and overall satisfaction with their stay in the hospital. The responses obtained allowed us to identify a group of key factors that patients consider most important (Figure 1). The friendliness of medical staff came first—as many as 82.9% of respondents indicated this element as particularly important. This was followed by the availability of specialists to ask questions and obtain advice (75.4%), specialist hospital equipment (59.8%) and the speed of healthcare services (54.5%). Patients also highly rated outstanding specialists (53.7%), a sense of safety (54.4%) and the overall cleanliness of the facility (52.6%). These factors clearly indicate that the professionalism of staff, organisational efficiency and customer service culture are of key importance to patients and not just technical or infrastructural issues. Furthermore, logistical factors and amenities such as ease of access (37.4%), access to sanitary facilities in the ward (37.3%), signage and intuitive navigation around the hospital (40.1%), and the quality of meals (34.7%) and short queues (31.1%) were included. Technical elements such as Internet access (11.1%), free television (12.2%) and quality certificates (8.1%) were less important. For women, the following factors are more important than they are for men: access to specialists (79.6% vs. 70.0%; *p* = 0.002), outstanding specialists (58.0% vs. 48.0%; *p* = 0.006), friendly staff towards patients (85.8% vs. 79.0%; *p* = 0.012), specialist equipment (65.6% vs. 52.0%; *p* < 0.001), fast healthcare services (58.2% vs. 49.5%; *p* = 0.016), a sense of security (59.8% vs. 47.1%; *p* < 0.001), short queues (34.2% vs. 27.0%; *p* = 0.034) and efficient telephone registration (17.5% vs. 12.0%; *p* = 0.034). These results clearly indicate that, from the patients’ perspective, the image of a hospital is primarily built on the quality of contact with staff, the availability of competent doctors and the organisational efficiency of healthcare services. Aesthetic qualities and additional amenities are only assessed in the second instance.

**Figure 2:**
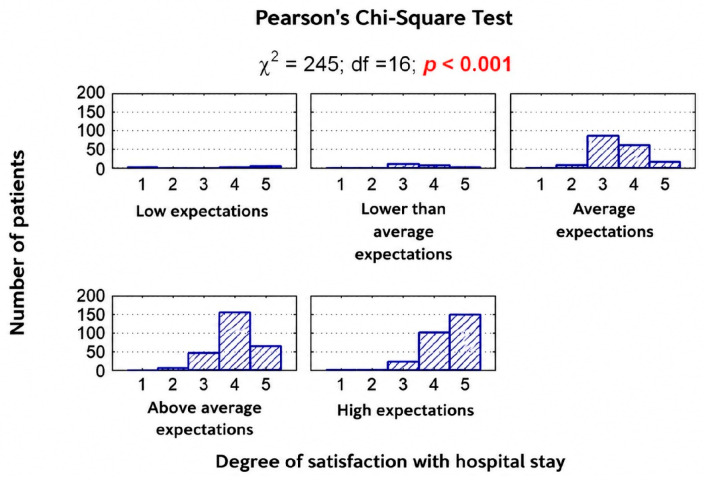
Number of patients in groups differing in terms of their expectations of a given hospital and their satisfaction with their stay there, and the results of the independence test.

**Figure 3:**
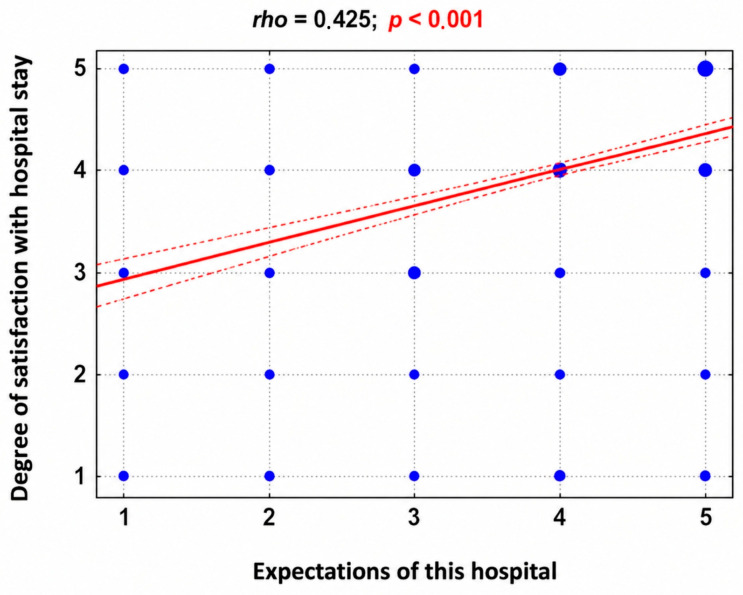
Correlation diagram between the degree of satisfaction with the hospital stay and expectations towards the hospital, and the value of Spearman’s rank correlation coefficient rho.

Open-ended responses provided additional context for the quantitative findings. Patients’ spontaneous associations with the hospital most often referred to staff professionalism, kindness, empathy, good care, trust, cleanliness, organization, comfort of stay, and safety. Negative associations concerned outdated infrastructure, overcrowding, poor sanitary conditions, waiting times, and administrative difficulties. In oncology-related wards, some responses also reflected the emotional burden of hospitalization, including fear, pain, helplessness, and uncertainty. These responses supported the quantitative results by showing that hospital image was shaped by both interpersonal and organizational factors, as well as by the clinical context of hospitalization. Patients rated their expectations of the hospital before treatment and then separately assessed their satisfaction with the hospital stay based on their actual experience of care, using a 1–5 scale. Patients proved to be quite satisfied with their stay in the hospital, which was confirmed by the fact that, if necessary, they would recommend the hospital to a family member or friend (42.0%—‘definitely yes’; 37.9%—‘rather yes’). In addition, a correlation between patients’ expectations and their satisfaction with their hospital stay was examined (Figure 2 and Figure 3).

A significant positive correlation was observed between the level of patients’ expectations towards a given hospital and their level of satisfaction with their stay at that facility (rho = 0.425, *p* < 0.001).

## 4. Discussion

The results of this study indicate that the image of public healthcare facilities in the opinion of patients is shaped primarily by factors related to the quality of interpersonal relations, the availability of specialists and the organization of care. In the analysed study, the factors that most frequently influenced patient satisfaction were the friendliness of staff (82.9%), the availability of specialists (75.4%) and a sense of security (54.4%). Infrastructure and technological elements, such as quality certificates or Internet access, were of much lesser importance. In interpreting these findings, it is important to distinguish between patient experience and patient satisfaction. Patient experience refers to the actual interactions and processes encountered by patients during hospitalization, including communication with staff, access to information, organizational efficiency, and perceived safety. Patient satisfaction, in contrast, reflects the patient’s subjective evaluation of whether these experiences met their prior expectations and individual needs. In the present study, patient experience was reflected in items concerning communication, staff attitudes, access to specialists, safety, and organizational aspects of care, whereas patient satisfaction was assessed through patients’ overall evaluation of their hospital stay and willingness to recommend the facility [18].

The open-ended responses provided additional context for the quantitative findings. Patients’ spontaneous associations with the hospital confirmed that hospital image is shaped by interpersonal factors, such as professional care, staff kindness, empathy, safety, and trust, as well as by organizational and infrastructural aspects, including cleanliness, comfort of stay, overcrowding, sanitary conditions, and waiting times. In oncology-related wards, associations also reflected the emotional burden of illness, including fear, pain, helplessness, uncertainty, and associations with death. These findings suggest that the patient-perceived image of a hospital is formed through the combined influence of relational, organizational, material, and clinical-context factors.

These conclusions are consistent with studies conducted in various European countries, which indicate that the success of implementing patient-centred care depends largely on the attitudes of staff rather than on technology or administrative standards [19]. It is not so much the infrastructural or technical elements that play a key role but rather the competence, kindness and empathy of medical staff [20].

The results confirm the concept previously postulated in the literature, according to which the subjective assessment of the quality of healthcare services results from the patient’s direct experience and interaction with staff and not solely from objective parameters of service provision [21]. The positive correlation between expectations and satisfaction observed in the present study (rho = 0.425, *p* < 0.001) is confirmed by other studies. Ramli and coauthors have shown that high expectations lead to a positive assessment of a hospital if the facility is able to meet those expectations, especially when the patient experiences high-quality communication and professional care [22]. The classic concept of Parasuraman, Zeithaml and Berry also indicates that satisfaction depends on the difference between expectations and the perception of service quality and that well-functioning facilities are able to build a positive image despite systemic limitations [23]. Research conducted in Italy has shown that patients with higher expectations show greater satisfaction if their sense of safety and the quality of interpersonal relationships are met, and as many as 94.5% of respondents rated the safety of hospitals positively [24]. In the analysed study, patients considered the most important factors influencing their satisfaction with their stay to be staff friendliness (82.9%), the availability of specialists (75.4%) and a sense of safety (54.4%). This finding is consistent with the findings of Gadalean, Cheptea and Constantin, who showed that the emotional climate of the ward—including communication style, courtesy and empathy—significantly influences the patient’s overall perception of the hospital [25].

In the context of sources of information about medical facilities, the results of this study clearly indicate the dominance of informal communication—primarily recommendations from acquaintances (34.1%) and previous personal experiences. Similar conclusions were drawn by Dziubaszewska and colleagues, who noted that patients value second-hand information more than media messages do, which are often perceived as incomplete or biased [26].

Comparable findings come from studies conducted in Central and Eastern European countries, including Poland, where dissatisfaction with the healthcare system as a whole coexists with a high level of satisfaction with specific facilities—especially when patients have had direct contact with empathetic staff [27]. Borowska et al. also emphasized that patients in Poland show limited trust in the healthcare system as institutions but, at the same time, express strong loyalty toward individual hospitals with which they have had positive experiences [28]. This suggests that microlevel relationships (patient–facility) can offset the generally negative image of the system. This is further confirmed by an analysis of patient statements regarding the media—public messaging was largely unknown or rated neutrally (77.9% of respondents had not encountered media materials about their hospital). Those who had assessed the content as accurate or moderate. Research conducted in China has shown that hospital reputation plays a key role in shaping patient satisfaction, surpassing the importance of treatment costs [29]. The importance of online information searches—as the most frequently indicated channel—has also been confirmed by research conducted in Syria, which revealed that hospital image mediates the relationships among cost, service quality, and patient satisfaction [8].

Based on the findings, several practical implications can be indicated. Hospital managers should systematically monitor patient feedback not only through satisfaction surveys but also through open-ended comments, as these may reveal aspects of care that are not fully captured by closed-ended questions. Particular attention should be paid to communication procedures, the clarity of information provided to patients, and the availability of staff for questions during hospitalization. Hospitals should also regularly assess organizational and environmental factors, such as signage, waiting times, cleanliness, sanitary conditions, and comfort of stay, because these elements contribute to the everyday patient experience and may influence institutional image. In addition, hospital communication strategies should take into account the importance of informal recommendations and online information, which patients use when forming opinions about healthcare facilities. In wards treating patients with severe or chronic diseases, image-building activities should also consider the emotional context of hospitalization and the need for psychological and relational support.

## 5. Conclusions

According to the results of the study, the image of a hospital in the eyes of patients is a complex construct shaped by both objective factors—such as infrastructure quality and service availability—and subjective elements, including personal experiences, recommendations from others, and the quality of interpersonal relationships with medical staff. Particularly important were staff kindness and empathy, access to information, and a sense of safety, which influenced the positive perception of the facility more than technical aspects did. These findings highlight the growing importance of organizational culture and communication in building patient trust and satisfaction.

The findings also showed that patients’ sociodemographic characteristics, previous contact with the facility, sources of information, and expectations were associated with how public hospitals were perceived. A positive relationship was observed between patients’ expectations and satisfaction with their hospital stay, which emphasizes the importance of consistency between prior expectations and actual experience. Overall, the study confirms that hospital image is shaped primarily by patient experience, interpersonal communication, and the perceived quality of care, indicating the importance of organizational culture and patient-centered communication in building trust and satisfaction.

## 6. Limitations of the Study

The research was conducted in eight public hospitals in Wrocław, which may limit the generalizability of the findings to other regions of Poland, rural areas, private facilities, or healthcare systems with different organizational characteristics. However, the inclusion of several hospitals within one large urban area allowed us to capture the local and institution-specific context of hospital image formation.

The study used a self-developed questionnaire. This approach was adopted because, at the time of data collection, there was no standardized Polish instrument that comprehensively assessed the patient-perceived image of public hospitals together with related factors such as general perception of the healthcare system, sources of information, media perception, previous hospital experience, expectations, and satisfaction with hospitalization. The questionnaire was developed on the basis of a literature review and the study objective, and was pilot-tested among hospitalized patients to improve the clarity and comprehensibility of selected questions. Nevertheless, future studies should further develop and validate standardized tools for assessing the patient-perceived image of hospitals.

All data were self-reported, which may have introduced recall bias, subjective interpretation of questions, and social desirability bias. Patients’ assessments may have been influenced by their current health status, emotions during hospitalization, previous healthcare experiences, or concerns about evaluating the hospital while still receiving care. Although participation was voluntary and anonymous, and patients were informed that the researcher was not a hospital employee, self-reported bias cannot be fully excluded.

The exact number of patients who refused to participate was not systematically recorded. Data collection was organized around obtaining a comparable number of completed questionnaires from each hospital, with an intended target of approximately 100 respondents per hospital, rather than calculating a formal response rate. Therefore, the precise refusal rate and response rate could not be determined.

Finally, the cross-sectional design does not allow for causal inference. In addition, potential confounding factors, such as ward type, severity of illness, health status, reason for hospitalization, and length of stay, were not fully controlled in the analysis. Future studies should include hospitals from different regions and ownership types, systematically record response rates, and consider longitudinal or mixed-methods designs.

## Figures and Tables

**Table 1:** General characteristics of patients hospitalized in selected public hospitals in Wrocław.

Characteristic (Variable)	*n*	%
1. Gender:		
Women	445	(57.2%)
Men	333	(42.8%)
2. Age:		
*M* ± *SD*	53.2 ± 17.2	
*Me* [*Q*1; *Q*3]	57 [37; 67]	
*Min*–*Max*	15–90	
3. Education:		
None	5	(0.6%)
Elementary	27	(3.5%)
Middle school	3	(0.4%)
Occupational	166	(21.3%)
Secondary	290	(37.3%)
Higher	275	(35.3%)
No information	12	(1.5%)
4. Place of residence—Province:		
Lower Silesia	708	(91.0%)
Including Wroclaw	382	(50.4%)
other	70	(9%)
5. Patient’s professional status:		
Pupil/student	25	(3.2%)
Professionally active	361	(46.4%)
Homemaker	20	(2.6%)
Unemployed	28	(3.6%)
Pensioner	331	(42.5%)
No information	13	(1.7%)
6. Duration of hospital stay:		
1 day	96	(12.3%)
2–3 days	241	(31.0%)
4–6 days	198	(25.4%)
A week or more	230	(29.6%)
No information	13	(1.6%)
7. Has been treated in this hospital before:		
Yes	323	(41.5%)
No	445	(57.2%)
No information	10	(1.3%)

**Table 2:** Self-survey questions on opinions about the health care system.

Self-Survey Question	*n*	%
Where do you get your information about the health care system?		
(a) Television	324	(41.6%)
(b) Radio	50	(6.4%)
(c) Press	130	(16.7%)
(d) Internet	404	(51.9%)
(e) Family	294	(37.8%)
(f) Friends and acquaintances	341	(43.8%)
(g) Leaflets, posters, guides, brochures	54	(6.9%)
(h) Encyclopaedias, books, guides	17	(2.2%)
(i) Specialist studies	34	(4.4%)
(j) Doctors, nurses, pharmacists, other health professionals	242	(31.1%)
(k) From other sources (which ones?) (mostly own experience)	28	(3.6%)
Are you interested in information about the health care system appearing in the media? (Please mark only 1 answer)		
(a) Yes, I keep up to date with information on this subject (5 points)	137	(17.6%)
(b) I pay attention to medical information more often than other information (4 points)	94	(12.1%)
(c) Yes, I take some interest in it (3 points)	251	(32.3%)
(d) I accidentally come across this type of information (2 points)	183	(23.5%)
(e) I am rather not interested in this (1 point)	83	(10.7%)
(f) I am not interested in it at all (0 points)	30	(3.9%)
*M* ± *SD*	2.9 ± 1.4 3 [2; 4] 0 ± 5	
*Me* [*Q*1; *Q*3]		
*Min* ± *Max*		
What type of information about the health care system, in your opinion, is most common in the media? (Please mark max. 3 answers)		
(a) Disasters, accidents	307	(39.5%)
(b) Scientific breakthroughs	149	(19.2%)
(c) Post-operative complications, medical errors	215	(27.6%)
(d) Local success stories of health care professionals and facilities	101	(13.0%)
(e) Corruption scandals in the medical community	83	(10.7%)
(f) Changes in the organisation of the health care system	170	(21.9%)
(g) Protests by the medical community	269	(34.6%)
(h) Drug reimbursement	140	(18.0%)
(i) Epidemiological data	9	(1.2%)
(j) Free preventive examinations	70	(9.0%)
(k) Waiting times for specialist services	201	25.8%)
(l) Financial problems of the health care system	287	(36.9%)
(m) Ranking of best medical facilities	30	(3.9%)
(n) Other	12	(1.5%)
Do you find the media to be a reliable source of information?		
(a) Yes (4 points)	60	(7.7%)
(b) Rather yes (3 points)	246	(31.6%)
(c) Difficult to say (2 points)	220	(28.3%)
(d) Rather not (1 point)	139	(17.9%)
(e) No (0 points)	113	(14.5%)
*M* ± *SD*	2.0 ± 1.2 2 [1; 3] 0–4	

**Table 3:** Self-survey questions on opinions about the relevant hospital.

Self-Survey Question	*n*	%
Have you seen any information in the media about the hospital where you are currently being treated?		
Yes	172	(22.1%)
No	606	(77.9%)
What had the greatest influence on your choice of the hospital where you are currently staying? (You may select up to 2 answers)		
(a) Specific media information about the hospital	21	(2.7%)
(b) General information appearing in the media	23	(3.0%)
(c) Opinion of family/friends	265	(34.1%)
(d) Previous personal experience	148	(19.0%)
(e) I was brought to the hospital by ambulance	106	(13.6%)
(f) The hospital is closest to my place of residence	176	(22.6%)
(g) Short waiting time for admission	156	(20.1%)
(h) Other	198	(25.4%)
In your opinion, what kind of information about this hospital appears in the media? (You may select more than one answer.)		
(a) I have not come across any information about this hospital in the media.	499	(64.1%)
(b) They fully reflect the actual situation—they show both the hospital’s achievements and negative events.	91	(11.7%)
(c) They are often consistent with the facts.	95	(12.2%)
(d) They are not substantive—journalists do not exhaust the subject.	40	(5.1%)
(e) The information presented shows the hospital only in superlatives.	21	(2.7%)
(f) They omit the positive aspects of the hospital’s activities, publicising only the negative ones.	26	(3.3%)
(g) They are sensationalist in nature, exaggerating crises, which puts the hospital in a negative light.	19	(2.4%)
(h) Other	17	(2.2%)
Have you ever looked for reviews about this hospital before?		
Yes	343	(44.1%)
No	435	(55.9%)
Where did you look for information and opinions about this hospital? N = 343		
(a) Among friends/neighbours	164	(47.8%)
(b) Among family members	98	(28.6%)
(c) In the Internet	160	(46.6%)
(d) In newspapers	10	(2.9%)
(e) Other sources	21	(6.1%)

## Data Availability

The original data presented in the study are openly available in https://katalog.bg.umw.edu.pl/site/recorddetail/0093100813374 (accessed on 30 October 2025).
